# Altered psychobiological reactivity but no impairment of emotion recognition following stress in adolescents with non-suicidal self-injury

**DOI:** 10.1007/s00406-022-01496-4

**Published:** 2022-10-06

**Authors:** Julian Koenig, Alexander Lischke, Kay Bardtke, Anna-Lena Heinze, Felix Kröller, Rike Pahnke, Michael Kaess

**Affiliations:** 1 Department of Child and Adolescent Psychiatry, Centre for Psychosocial Medicine, University of Heidelberg, Heidelberg, Germany; 2 University Hospital of Child and Adolescent Psychiatry and Psychotherapy, University of Bern, Bern, Switzerland; 3 Faculty of Medicine and University Hospital Cologne, Department of Child and Adolescent Psychiatry, Psychosomatics and Psychotherapy, University of Cologne, Cologne, Germany; 4 Department of Psychology, Medical School Hamburg, Hamburg, Germany; 5 Department of Sport Science, University of Rostock, Rostock, Germany

**Keywords:** Stress, Emotion recognition, Borderline personality disorder, Cortisol, Alpha-amylase

## Abstract

Impairments in both stress regulation and emotion recognition have been associated with borderline personality disorder (BPD) and non-suicidal self-injury (NSSI). Although it has been proposed that emotion recognition deficits particularly emerge during stress, this hypothesis has not been fully investigated. Adolescents with and without NSSI performed emotion recognition tasks before and after the employment of the Trier Social Stress Test (TSST). The psychobiological stress response was captured with psychological self-reports (affect, stress and dissociation), physiological recordings (heart rate, HR, and heart rate variability, HRV) and endocrinological sampling of saliva (cortisol and alpha-amylase). Mixed-linear models were applied to analyze stress-induced changes in emotion recognition performance and respective stress response measures. The TSST elicited altered psychobiological stress responses in adolescents with NSSI: A more pronounced decrease in positive affect, a more pronounced increase in negative affect, a less pronounced increase in HR, a less pronounced decrease in HRV and a more pronounced increase in alpha-amylase throughout the stress induction than adolescents without NSSI. Stress responses (dissociation, negative affect, cortisol and HR) differed as a function of BPD severity on a continuum, illustrating greater reactivity on self-reports but decreased biological responsiveness in those with greater BPD severity. Stress induction had similar effects on emotion recognition in adolescents with and without NSSI. Recognition sensitivity and recognition speed equally increased, in the absence of any differences in recognition accuracy. In contrast to prominent propositions, psychosocial stress does not appear to account for impaired emotion recognition across the BPD spectrum.

**Supplementary Information:**

The online version contains supplementary material available at 10.1007/s00406-022-01496-4.

## Introduction

Borderline personality disorder (BPD) is a severe mental disorder that is associated with a substantial morbidity and immense health costs [1, 2]. Most research has focused on adults with BPD whose pathology is mainly characterized by affective instability and impulsive behavior [3]. Anger outbursts, aggressive actions, self-injurious and suicidal acts are core features of BPD pathology. These core features can also be found among adolescents [4], indicating that affective instability and impulsive behavior are part of a wider BPD spectrum [5, 6]. One of the most prevalent clinical precursors of BPD in adolescents is non-suicidal self-injury (NSSI) [7]. Adolescent BPD is frequently characterized by an over-representation of acute symptoms—such as NSSI [8]. Recent research suggests that affective instability and impulsive behavior paves the way from such subclinical BPD pathology in adolescence to clinical BPD pathology in adulthood [8]. Therefore, studying adolescents with NSSI across the spectrum of BPD pathology may offer important insights into the developmental pathways of full-blown BPD.

Affective instability and impulsive behavior of adolescents and adults with NSSI and BPD often manifest itself during social interactions [9, 10], indicating the processing of social cues is impaired. Adults and adolescents with BPD show various impairments in social cue processing, in particular during the identification and discrimination of emotional expressions [11, 12]. Impairments in emotion recognition, thus, may accelerate the development of subclinical BPD pathology in adolescence into clinical BPD pathology in adulthood [5, 13].

Although a number of studies investigated emotion recognition in adults with BPD, these studies revealed inconsistent findings [11, 12]. Some studies found adults with BPD to be less accurate in emotion recognition than adults without BPD, in particular during the processing of negative emotions [14–19]. Adults with BPD had difficulties in identifying negative emotions [14, 15, 17, 18] and tended to misclassify other emotions as negative emotions [15, 16, 18, 19]. Other studies were unable to find such differences in recognition accuracy between adults with and without BPD. Adults with BPD either showed similar [20] or enhanced recognition accuracy during emotion processing [18, 21, 22].

Studies investigating emotion recognition in adolescents with BPD also revealed inconsistent findings [12]. Some studies found adolescents with BPD to be less accurate in emotion recognition than adolescents without BPD [23]. Adolescents with BPD had more difficulties in identifying emotions, albeit only during the processing of low and not high intensity emotions [23]. Other studies were unable to illustrate such differences in recognition accuracy between adolescents with and without BPD. Adolescents with BPD either showed similar [24] or enhanced recognition accuracy during emotion processing [25].

Overall, studies on emotion recognition in adolescents and adults with BPD are characterized by considerable heterogeneity of findings. A meta-analytic review of these findings suggest that there are subtle rather than frank impairments in emotion recognition across the BPD spectrum [12]. Common theories propose that these impairments are most likely to emerge during social encounters that are characterized by psychosocial stress [11]. Psychosocial stress is thought to alter arousal levels in patients with BPD in a way that differentially affects the processing of low and high intensity emotions in social contexts: Low intensity emotions are believed to be recognized with higher than normal accuracy and high intensity emotions are believed to be recognized with lower than normal accuracy. Indeed, there is substantial evidence on altered psychobiological stress reactivity both in BPD and NSSI. BPD patient’s show blunted cortisol secretion during psychosocial stress [26]. Similar, there is converging evidence for an attenuated cortisol response to stress in individuals with NSSI [27]. In addition to altered functioning of the hypothalamic–pituitary–adrenal (HPA) axis, patients with BPD and/or NSSI show altered autonomic nervous system (ANS) responses to stress, although relatively few studies addressed ANS stress reactivity in these patients. Individuals with BPD and/or NSSI seem to be characterized by an ANS profile reflecting greater sympathetic dominance [27, 28]. Although these propositions appear to be plausible interfering with emotion recognition during states of altered bodily arousal, the propositions have rarely been tested on empirical grounds. Preliminary studies in adults with BPD revealed limited support in favor of these propositions [29, 30], indicating a need to replicate and extend the findings from these studies.

The present study investigated for the first time how psychosocial stress (including the respective psychobiological measures of reactivity) affects emotion recognition in adolescents presenting with NSSI across the spectrum of BPD pathology. Utilizing a dimensional approach to BPD pathology [6], adolescents showing core features of BPD pathology such as affective instability and impulsive behavior were recruited. Given that NSSI is a precursor of adult BPD [31], recruitment focused on adolescents with NSSI. Adolescents with and without NSSI completed a series of emotion recognition tasks before and after the induction of psychosocial stress. Following contemporary theories on stress-induced alterations in emotion recognition [11], adolescents with NSSI were expected to show subtle impairments in emotion recognition following stress induction.

## Methods

### General procedures and participant flow

The ethics committee of the Medical Faculty, Heidelberg University approved the study (Study ID: S-685/2015). Adolescents with NSSI were recruited at the specialized outpatient clinic for risk taking and self-injurious behavior (AtR!Sk [32]) at the Clinic for Child and Adolescents Psychiatry, Heidelberg University. Adolescents without NSSI were recruited via public advertisement in the Heidelberg catchment area. All adolescents were initially screened in person or via telephone for inclusion in the trial according to pre-defined inclusion and exclusion criteria. In order to be included, adolescents had to be between 13 and 17 years of age and female. Male adolescents were excluded to rule out potential sex-dependent differences in measures of interest. Adolescents were not included if they had difficulties in reading or understanding German language; took glucocorticoid medication; were pregnant; reported any neurological disorder; reported acute psychotic symptoms; reported acute suicidality; reported substance dependence; or had a body mass index (BMI) below 17.5 or above 30 kg/m^2^. Adolescent with NSSI had to meet diagnostic criteria for NSSI according to DSM-5 (see Appendix). Healthy adolescents were only included if not fulfilling criteria for any lifetime NSSI and current psychiatric disorder.

**Fig. 1:**
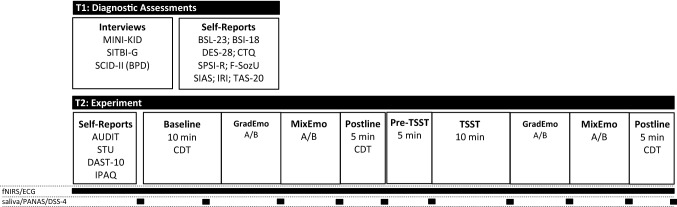
Study Design; *MINI-KID* Mini-International Neuropsychiatric Interview for Children and Adolescents, *SITBI-G* Self-Injurious Thoughts and Behavior Interview, *SCID-II* Structured Clinical Interview for DSM-IV-Axis II BPD module, *BSL-23* Borderline Symptom List Short-Form, *BSI-18* Brief Symptom Inventory, *DES-28* Dissociative Experience Scale, *CTQ* Childhood Trauma Questionnaire, *SPSI-R* Social Problem Solving Inventory, *FSozU* Social Support Questionnaire, *SIAS* Social Interaction Anxiety Scale, *IRI* Interpersonal Reactivity Index, *TAS-20* Toronto Alexithymia Scale, *AUDIT* Alcohol Use Disorders Identification Test, *STU* Smoking And Tobacco Use Questionnaire, *DAST-10* Drug Abuse Screening Questionnaire, *IPAQ* International Physical Activity Questionnaire, *CDT* Color Detection Task, *TSST* Trier Social Stress Test, *fNIRS* functional near-infrared spectroscopy, *ECG* electrocardiography, *PANAS* Positive and Negative Affect Schedule, *DSS-4* Dissociation–Tension Scale

A total of *n* = 180 consecutive patients and *n* = 63 controls were screened for inclusion. *N* = 37 patients were invited for participation in the study, of which *n* = 7 dropped out. *N* = 31 adolescents without NSSI were included in the study, of which *n* = 1 dropped out during the experiment. The final sample comprised *n* = 30 adolescents with NSSI and *n* = 30 healthy adolescents without NSSI. Reasons for exclusion and drop-out are provided in the Supplementary Material. All adolescents and their legal guardians provided written informed consent before inclusion in the study. Participation in the study comprised two appointments following screening. At T1, all diagnostic assessments (interviews and questionnaires) were conducted. At T2, the actual experiment took place as detailed below. Participants received an allowance of 40€ (20€ for each appointment) for participation. An overview of the study design is provided in Fig. 1.

### Clinical assessments and self-reports (T1)

Following an initial screening, participants were informed about the study details. In case written informed consent was obtained, participants were assigned a study ID and invited to a first appointment including all clinical assessments (T1). At T1, participants provided basic sociodemographic information before completing several clinical interviews. All assessments were parallelized across groups. Clinical interviews included the German version of the *Mini-International Neuropsychiatric Interview for Children and Adolescents* (M.I.N.I- KID 6.0; [33]), the German version of the *Self-Injurious Thoughts and Behavior Interview* (SITBI-G; [34]); the German version of the *Structured Clinical Interview for DSM-IV-Axis II* BPD module (SCID-II; [35]). Further, all participants completed the following self-reports: the *Borderline Symptom List Short-Form* (BSL-23; [36]); the *Brief Symptom Inventory* (BSI-18 [37]); the *Dissociative Experience Scale* (DES-28; [38]; the *Childhood Trauma Questionnaire* (CTQ [39]); other instruments—not reported in the present Paper—included the *Social Problem Solving Inventory* (SPSI-R; [40]); the brief form of the *Social Support Questionnaire* (F-SozU [41]); the *Social Interaction Anxiety Scale* (SIAS; [42]); the *Interpersonal Reactivity Index* (IRI; [43]); and the *Toronto Alexithymia Scale* (TAS-20 [44]). All interviews and self-reports were computerized using *LimeSurvey*.

### Experiment (T2)

All appointments for T2 were scheduled in the afternoon (past 1 pm). Time stamps for all events were recorded on computerized paper (TeleForm–Electric Paper). Participants were invited to the lab. Their weight and height were recorded before they completed several computerized questionnaires during an acclimatization phase. Questionnaires included the *Alcohol Use Disorders Identification Test* (AUDIT; [45]); a short version of the *Smoking And Tobacco Use* (STU) *Questionnaire*, based on the respective WHO instrument; the *Drug Abuse Screening Questionnaire* (DAST-10; [46]), and the sort version of the *International Physical Activity Questionnaire* (IPAQ; [47]). Following the questionnaires, participants were equipped with recording sensors (detailed below) for the continuous monitoring of prefrontal cortex oxygenation using functional near-infrared spectroscopy (fNIRS^1^) and electrocardiography (ECG). Following a 10-min baseline recording of physiological data, using a computerized *Color Detection Task* (CDT; [48]), participants completed the first set of emotion recognition tasks. Each set included two tasks: one on gradually expressed emotions (*GradEmo*) and one on mixed expressions of emotion (*MixEmo*). Following the completion of the first set of tasks, a 5-min postline was recorded, after which participants were instructed about the following stress-induction paradigm. All participants underwent the Trier Social Stress Test (TSST [49]). The TSST is a well-evaluated standardized psychosocial stress protocol, adapted for the underage population, comprising a mock school/university interview and a mental arithmetic task (5 min each) performed in front of an audience of two auditors while being videotaped. Importantly, the audience is non-responsive to the participant during the interview. In the present study, participants were instructed to prepare for the interview 5 min before the start of the interview. Immediately following the TSST, participants completed the second set of emotion recognition tasks, after which another 5-min postline was recorded and participants were debriefed.

### Emotion recognition

**Fig. 2:**
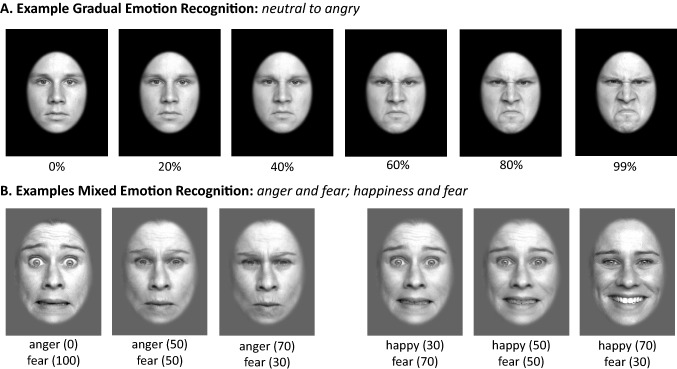
Examples of the visual stimuli used; **A** illustrated are 6 steps of a morph sequence from neutral to angry; **B** illustrated are two examples for the presentation of mixed emotions (left: anger and fear; right: happiness and fear)

Two different emotion recognition tasks were used. The first task (*GradEmo*) used gradually increasing emotions, in the second task (*MixEmo*) emotions were mixed. Stimuli for both tasks were taken from the FACES database, a database containing color pictures of faces with different emotional expressions [51]. Following an established procedure [52], the pictures were converted to greyscale and masked with an epileptic frame that removed head hair from the faces. All tasks were programmed using PsychoPy (version: 1.84; [50] in two versions (A/B) for repeated presentation, using distinct sets of stimuli. The order of tasks was kept constant (GradEmo first), while the order of versions was randomized. Examples of the visual stimuli used, are provided in Fig. 2.

*GradEmo*: Each version of the task (A and B) comprised the presentation of 24 faces expressing four emotions (angry, disgusted, fearful, happy). Each stimulus was manipulated to generate a morph sequence of 100 pictures, morphing from *neutral* to one of the emotional expressions, resulting in a total of 24 morph sequences per version of the task. Morph sequences were generated using WinMorph (Satish Sampath, DebugMode, Version 3.01). Following an instruction screening, participants were presented with the morph sequences, illustrating each picture for 160 ms, gradually increasing the emotional expression (0–99%). The presentation of a fixation cross (2 s) preceded each new morph sequence. When the mouse key was pressed, the morph sequence stopped and participants were asked to select the category of emotional expression (angry, disgusted, fearful, happy) within 10 s. The GradEmo task comprised pictures from 3 females and 3 males, each showing the 4 distinct emotions. Version A and B of the task did not include the same actors or faces. Within each version of task, the presentation of pictures was randomized, not presenting same-sex pictures subsequently. The response time (time until recognition of emotional expression and selection of one emotion) in ms, the level of emotion expression at response in percent, and the selected category (correct responses) were recorded.

*MixEmo*: Each version of the task comprised 60 static pictures, presenting mixed emotions of the following combinations: angry–happy; angry–fearful; fearful–happy. Pictures of mixed emotions were generated by blending two different emotions (expressed by the same actor) using WinMorph (Satish Sampath, DebugMode, Version 3.01). For each pair of emotions, 5 different mixes (with varying levels of blending intensity) were generated: 30–70, 40–60, 50–50, 60–40 and 70–30%. For each version of the task, pictures from 2 female and 2 male actors were used. Again, the presentation of a fixation cross (2 s) preceded each new picture. Participants had 5 s for each trial to indicate the dominant emotion, expressed in the respective picture. The response time (time until recognition of emotional expression and selection of one emotion) in ms, and the selected category (correct responses) were recorded.

### Repeated self-reports

During the experiment, parallelized with the collection of saliva (see below), participants provided self-reports on the computerized *Positive and Negative Affect Schedule* (PANAS [53]) the *Dissociation–Tension Scale* (DSS-4; [54]) and a single item on current stress rated on a visual analog scale [VAS; 100 mm].

### Neurobiological measures

Reporting of electrocardiography (ECG) processing and analyses adheres to the *Guidelines for Reporting Articles on Psychiatry and Heart Rate Variability* (GRAPH [55]). ECG were recorded using an ekgMove 3 sensor (movisens GmbH, Karlsruhe, Germany) attached to participants’ chest at the base of the sternum using a flexible belt with two integrated electrodes that were watered before the recording. ECG signals were recorded at a sampling rate of 1024 Hz. Data were visually inspected after every recording using the unisens viewer (version: 2.0) and saved in the csv format. ECG data were further processed in Kubios HRV 3.0 Premium [56]. R-Peak detection was manually corrected and artifacts were removed. On average 98.73% of data (SD = 4.81) were artifact free. Details are provided in the Supplementary Material. Smoothing priors were selected as detrending method (*λ* 500) for IBI data. Kubios output was saved in the txt format for later automated readout of corrected inter-beat-intervals (IBIs) and analysis of heart rate variability (HRV) in R [57]. IBIs corresponding to a mean HR < 30 or > 200 bpm were discarded and data were segmented in accordance with experimental conditions. The square root of the mean squared difference of successive IBIs (RMSSD) measured in ms, a time-domain measure of heart rate variability (HRV) indexing vagal activity [58] and the mean heart rate (HR), were calculated for 11 segments for each participant during T2. Missing data, duration and artifact-rate by segment are available upon request. NIRS and ECG data during baseline (10 min), and both postline (5 min each) were recorded while participants completed a CDT. Based on existing recommendations [48], such *vanilla baseline* is designed to be minimally demanding, asking participants to count the number of times a rectangle on the computer screen changed to a designated color, providing the count at the end of the task. The color of the rectangle (yellow, white, red, blue, green or purple; randomized) changed every 10 s. The color and times that the respective color appeared were randomly determined. Across the study, in 82.42% of the cases, participants provided the exact correct number. The start and end-time of the CDT was tracked to synchronize all physiological recordings to the task condition. The CDT was programmed using PsychoPy (version: 1.84: [50]. The CDT and all other neuropsychological tasks were displayed on a FLATRON IPS231 (LG) computer screen with a resolution of 1920 × 1080 pixels. A total of 10 saliva samples were collected by having participants chew on a cotton role (Salivettes; Sarstedt, Numbrecht, Germany) for > 1 min, while completing the self-reports on current stress and mood (see Fig. 1). Saliva samples were collected before (S1) and following (S2) the first baseline; between the first set of the two emotion recognition tasks (S3); after the second emotion recognition task (S4); following the first postline (S5); following the TSST preparation phase (S6); following the TSST (S7); between the second set of the two emotion recognition tasks (S8); after the final emotion recognition task (S9); and following the second postline (S10). Saliva samples were labeled and stored at – 20 °C until assay conducted at the Department of Psychology, Technical University of Dresden. Technical documentation on the in-house assays used is provided elsewhere [59, 60]. *Cortisol* and *α-amylase* were determined from each sample. Information on complete data by group, time of assessment and hormone is available upon request.

### Statistical analyses

Differences between groups on sociodemographic and clinical variables were analyzed using chi-square (categorical variables) or *t* tests (continuous variables), respectively. Task data on emotion recognition were analyzed using multilevel mixed-effects generalized linear models for binominal data (dichotomous outcomes) or multilevel mixed-effects linear regression (continuous outcomes). Group (patients versus controls) and condition (no-stress versus stress) as well as their interaction were addressed as fixed effects. The participants’ ID was entered as random effect. Self-reports (stress, positive and negative effect, dissociation) as well as physiological data (HR, HRV, cortisol, α-amylase) were analyzed using multilevel mixed-effects linear regression with time (segment or time of measurement), group and condition as fixed effects and the participants’ ID as random effect. In additional to models addressing main and interaction effects of group allocation and time (segment or time of measurement), models were repeated utilizing a dimensional approach to BPD severity based on both BSL-23 scores and BPD criteria (SCID-II) in secondary analyses. Only the respective interaction terms were addressed (TIME by SEVERITY) and checked for consistency across both measurement modalities (BSL-23 and SCID-II). For the interpretation of the respective continues interactions that were consistent across both measures of BPD severity, margin plots at fixed levels of BPD severity (BPD criteria: 0 | 3 | 6 | 9; BSL-23: 0 | 1 | 2 | 3 | 4) were derived and are presented in the Supplementary Material. All analyses were performed using Stata (Version 15.1; StataCorp LP, College Station, TX, USA), at an *alpha* level of 0.05. All contrasts were Sidak corrected.

## Results

### Sample characteristics

Sociodemographic and clinical characteristics by group are provided in Table 1. Groups significantly differed on weight (*t*_(58)_ = 2.348, *p* = *0.022*), with patients reporting greater weight. Groups differed on all clinical variables, with patients reporting greater borderline symptom severity (BSL-23: *t*_(58)_ = 10.797, *p* < *0.0001*), global symptom severity (BSI-GSI: *t*_(58)_ = 9.059, *p* < *0.0001*); dissociative experiences (*t*_(58)_ = 6.003, *p* < *0.0001*), trauma severity (*t*_(58)_ = 5.452, *p* < *0.0001*), and endorsed more BPD criteria (*t*_(58)_ = 10.450, *p* < *0.0001*). *N* = 7 patients endorsed > 5 BPD criteria, fulfilling the diagnostic threshold (23.33%). On average, patients engaged in NSSI on 89.03 (SD: 144.90; 5–720) days in the past year. *N* = 15 patients (50%) reported a previous suicide attempt with a mean of 1.2 (SD: 1.6; 0–5) attempts in the past year. On average, patients reported 1.5 diagnoses (SD: 0.97; 0–3).

**Table 1:** Sociodemographic and clinical characteristics by group

	Adolescents with NSSI	Adolescents without NSSI	*p*
*N* (female %)	30 (100.00)	30 (100.00)	
Height, cm	165.83 (4.98)	167.03 (4.43)	0.328
Weight, kg	61.00 (11.01)	55.47 (6.73)	0.022
School, *n* (%)			0.672
Hauptschule	1 (3.33)	0 (0.00)	
Realschule	7 (23.33)	5 (16.67)	
Gymnasium	20 (66.66)	23 (76.67)	
Other	2 (6.66)	2 (6.67)	
BSL-23	1.83 (0.83)	0.15 (0.17)	< 0.0001
BSI-GSI	1.54 (0.75)	0.25 (0.19)	< 0.0001
DES	20.83 (13.63)	5.08 (4.56)	< 0.0001
CTQ	43.87 (15.05)	28.30 (4.26)	< 0.0001
BPD, cat	7 (23.33)	0.00 (0.00)	0.005
BPD, # criteria	3.53 (1.85)	0.00 (0.00)	< 0.0001
BPD fulfilled criteria, *n* (%)			
Paranoid ideas	9 (30.00)	0 (0.00)	< 0.0001
Intense anger	9 (30.00)	0 (0.00)	< 0.0001
Chronic emptiness	10 (33.33)	0 (0.00)	< 0.0001
Affective instability	21 (70.00)	0 (0.00)	< 0.0001
Suicidal behavior	28 (93.33)	0 (0.00)	< 0.0001
Impulsivity	6 (20.00)	0 (0.00)	< 0.0001
Identity disturbance	9 (30.00)	0 (0.00)	< 0.0001
Interpersonal unstable	16 (53.33)	0 (0.00)	< 0.0001
Avoid abandonment	6 (20.00)	0 (0.00)	< 0.0001
Comorbidity (ICD-10), *n* (%)			
F0X	0 (0.00)	0 (0.00)	
F1X	3 (10.00)	0 (0.00)	0.076
F2X	0 (0.00)	0 (0.00)	
F3X	17 (56.66)	0 (0.00)	< 0.0001
F4X	14 (46.66)	0 (0.00)	< 0.0001
F5X	1 (3.33)	0 (0.00)	0.313
F6X	6 (20.00)	0 (0.00)	0.010
F7X	0 (0.00)	0 (0.00)	
F8X	1 (3.33)	0 (0.00)	0.313
F9X	3 (10.00)	0 (0.00)	0.076

All values are means (M) and standard deviations (SD) in brackets unless otherwise indicated; *school*: After four years of elementary school the German school system branches into three types of secondary schools. The so-called *Hauptschule* (Secondary General School which takes five years after Primary School) prepares pupils for vocational training, whereas the *Realschule* (Intermediate Secondary School) concludes with a general certificate of secondary education after six years. Eight years of Gymnasium provide pupils with a general university entrance qualification

*Medication* multiple counts possible, data on doses and duration of intake available upon request, *BSL-23* Borderline Symptom List, *BSI-GSI* Brief Symptom Inventory Global Severity Index, *DES* Dissociative Experiences Scale, *CTQ* Childhood Trauma Questionnaire, *BPD* patients fulfilling criteria for BPD diagnosis (SCID-II) and number of fulfilled BPD criteria, *BPD fulfilled criteria* breakdown of fulfilled BPD criteria (partially fulfilled scored as not fulfilled)

### Stress induction: manipulation check

**Fig. 3:**
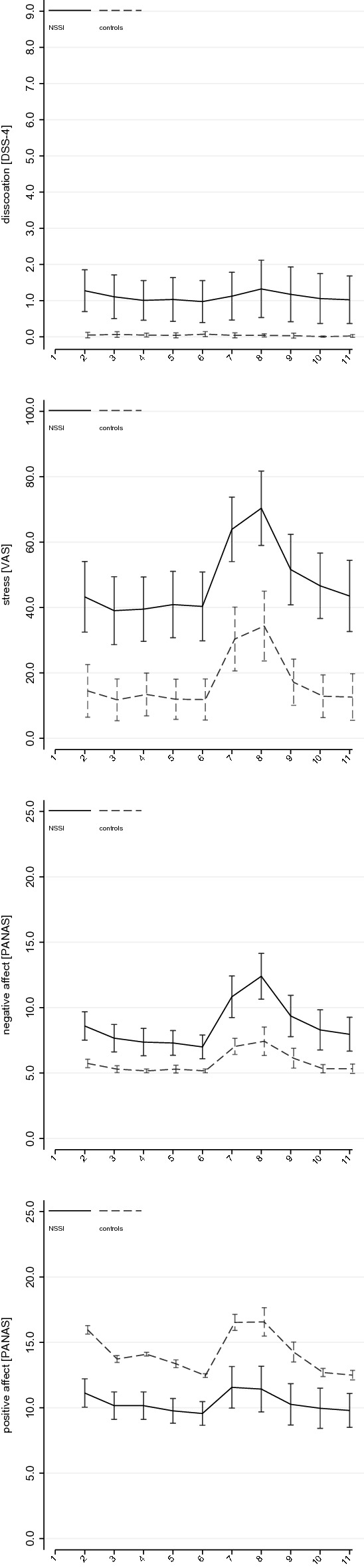
Effects of stress induction on self-reports; *DSS-4* Dissociation–Tension Scale, *VAS* visual analog scale,* NSSI* patients with non-suicidal self-injury

Mixed models based on GROUP allocation addressing effects on self-reports of dissociation (*χ*^2^_(19)_ = 33.60, *p* = *0.021*), stress (*χ*^2^_(19)_ = 362.13, *p* < *0.0001*), positive (*χ*^2^_(19)_ = 275.13, *p* < *0.0001*), and negative affect (*χ*^2^_(19)_ = 345.95, *p* < *0.0001*), all showed significant model fit. There were significant main effects of GROUP on reports of dissociation (*χ*^2^_(1)_ = 12.69, *p* = *0.0003*), stress (*χ*^2^_(1)_ = 32.21, *p* < *0.0001*), positive (*χ*^2^_(1)_ = 19.51, *p* < *0.0001*), and negative affect (*χ*^2^_(1)_ = 30.87, *p* < *0.0001*), indicating greater dissociation and stress, as well as decreased positive and increased negative affect in patients with NSSI compared to controls, independent of stress induction. There were significant main effects of TIME on self-reports of stress (*χ*^2^_(9)_ = 319.69, *p* < *0.0001*), as well as positive (*χ*^2^_(9)_ = 221.33, *p* < *0.0001*), and negative affect (*χ*^2^_(9)_ = 277.09, *p* < *0.0001*). Significant GROUP*TIME interactions were observed for positive (*χ*^2^_(9)_ = 34.29, *p* < *0.001*) and negative affect (*χ*^2^_(9)_ = 38.00, *p* < *0.0001*) only. Patients with NSSI showed a greater increase in negative affect following stress induction, whereas controls showed a greater increase in positive affect following stress induction. Findings are illustrated in Fig. 3. Continuous models on BPD severity all showed significant model fit independent of predicted outcome (self-reports). There was a significant TIME by SEVERITY interaction, consistent across measures (BSL-23 and SCID-II), in predicting self-reports of dissociation (BSL-23: *χ*^2^_(9)_ = 22.52, *p* = *0.007*; SCID-II: *χ*^2^_(9)_ = 20.35, *p* = *0.016*) and negative affect (BSL-23: *χ*^2^_(9)_ = 82.35, *p* < *0.0001*; SCID-II: *χ*^2^_(9)_ = 56.75, *p* < *0.0001);* but not stress (BSL-23: *χ*^2^_(9)_ = 27.70, *p* = *0.001*; SCID-II: *χ*^2^_(9)_ = 11.21, *p* = *0.262*) or positive affect (BSL-23: *χ*^2^_(9)_ = 13.67, *p* = *0.135*; SCID-II: *χ*^2^_(9)_ = 24.56, *p* = *0.010*) that showed inconsistent findings across measures of BPD severity. The respective findings are illustrated in Figure SM1 (dissociation) and Figure SM2 (negative affect).

**Fig. 4:**
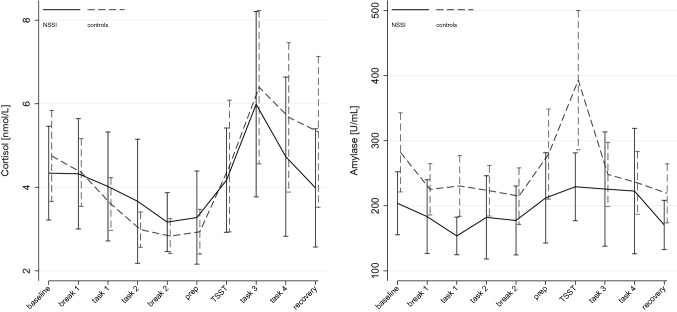
Effects of stress induction on saliva cortisol and α-amylase

**Fig. 5:**
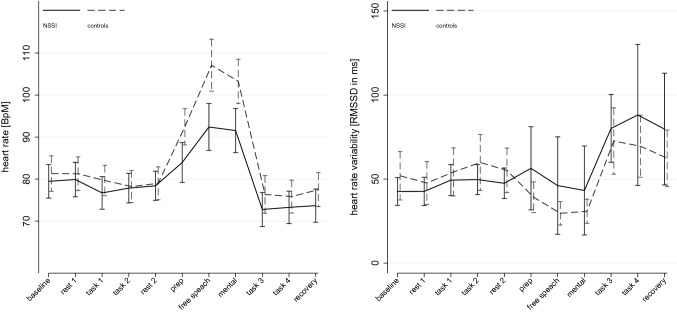
Effects of stress induction on heart rate and heart rate variability, *BpM* beats per minute, *RMSSD* root mean square of successive differences

Analyses of saliva cortisol (*χ*^2^_(19)_ = 103.96, *p* < *0.0001*) and α-amylase (*χ*^2^_(19)_ = 97.18, *p* < *0.0001*) showed significant model fit. There were no significant main effects of GROUP, but a significant main effect of TIME on cortisol (*χ*^2^_(3)_ = 94.07, *p* < *0.0001*) and α-amylase (*χ*^2^_(3)_ = 70.03, *p* < *0.0001*) – both increased following stress induction. α-amylase further showed a significant GROUP*TIME interaction (*χ*^2^_(3)_ = 23.86, *p* = *0.005*), indicating greater release in controls compared to patients with NSSI. Findings are illustrated in Fig. 4. Mixed-effect analyses on HR (*χ*^2^_(21)_ = 1090.65, *p* < *0.0001*) and HRV (*χ*^2^_(21)_ = 122.75, *p* < *0.0001*) showed significant model fit. There were no main effects of GROUP but TIME on HR (*χ*^2^_(10)_ = 1022.47, *p* < *0.0001*) and HRV (*χ*^2^_(10)_ = 104.33, *p* < *0.0001*). There were significant interactions of GROUP*TIME on both, HR (*χ*^2^_(10)_ = 74.18, *p* < *0.0001*) and HRV (*χ*^2^_(10)_ = 18.53, *p* = *0.047*), indicating different trajectories of HR and HRV between groups over time. Findings are illustrated in Fig. 5. Again, continuous models on BPD severity all showed significant model fit independent of predicted outcome (biological markers). There was a significant TIME by SEVERITY interaction, consistent across measures (BSL-23 and SCID-II), in predicting cortisol secretion (BSL-23: *χ*^2^_(9)_ = 17.15, *p* = *0.046*; SCID-II: *χ*^2^_(9)_ = 25.79, *p* = *0.002*) and HR (BSL-23: *χ*^2^_(9)_ = 44.16, *p* < *0.0001*; SCID-II: *χ*^2^_(9)_ = 51.15, *p* < *0.0001*), but not α-amylase (BSL-23: *χ*^2^_(9)_ = 8.62, *p* = *0.473*; SCID-II: *χ*^2^_(9)_ = 17.32, *p* = *0.044*) or HRV (BSL-23: *χ*^2^_(9)_ = 1.46, *p* = *0.999*; SCID-II: *χ*^2^_(9)_ = 2.84, *p* = *0.985*) that showed inconsistent findings across measures of BPD severity*.* The respective findings as a function of BPD severity are illustrated in Figure SM3 (cortisol) and Figure SM4 (HR).

### Emotion recognition task performance

Descriptive statistics on task performance by task outcome, group and time are given in Table 2.

**Table 2:** Descriptive statistics by task outcome, group and time

	Adolescents with NSSI						Adolescents without NSSI					
	No-stress			Stress			No-stress			Stress		
	*n*	Mean (SD)	Range	*n*	Mean (SD)	Range	*n*	Mean (SD)	Range	*n*	Mean (SD)	Range
Gradually expressed emotions												
Correctly classified [*n*]	30	20.2 (2.47)	14–23	30	20.7 (2.09)	16–24	30	20.9 (1.92)	16–24	30	20.67 (2.09)	17–24
Correctly classified [%]	30	84.60 (10.29)	59.33–95.82	30	86.25 (8.69)	66.67–100	30	87.08 (7.99)	66.67–100	30	86.11 (8.71)	70.83–100
Morph Intensity [%]	30	54.98 (16.91)	32.08–98.21	30	43.70 (13.37)	20.29–75.71	30	50.77 (9.25)	30.33–71.67	30	42.88 (9.53)	25.79–63.25
Morph time [sec]	30	8.73 (2.72)	5.04–15.71	30	6.91 (2.14)	3.18–12.04	30	8.05 (1.48)	4.78–11.40	30	6.78 (1.53)	4.04–10.04
Response Time [sec]	30	3.23 (0.58)	2.07–4.69	30	2.44 (0.41)	1.74–3.33	30	3.35 (0.77)	2.05–6.06	30	2.60 (0.50)	1.72–4.03
Morph Intensity [%] correct	30	54.21 (16.06)	31.47–98.05	30	43.03 (12.89)	19.29–71.33	30	50.16 (8.57)	31.00–70.71	30	42.34 (9.54)	25.47–63.96
Morph time/correct [sec]	30	8.60 (2.58)	4.94–15.68	30	6.81 (2.06)	3.00–11.34	30	7.95 (1.37)	4.90–11.24	30	6.70 (1.53)	3.98–10.15
Response Time [sec] correct	30	3.00 (0.51)	1.80–4.03	30	2.34 (0.37)	1.73–3.31	30	3.11 (0.61)	2.05–4.75	30	2.53 (0.47)	1.71–3.97
Mixed expressed emotions												
Correctly classified [*n*]	30	51.53 (3.90)	41–59	30	50.67 (3.97)	42–57	30	51.37 (5.69)	31–57	30	51.30 (4.24)	36–57
Correctly classified [%]	30	85.59 (6.50)	68.33–98.33	30	84.44 (6.61)	70–95	30	85.61 (9.48)	51.67–95	30	85.50 (7.06)	60–95
Response time [sec]	30	1.82 (0.39)	1.22–2.83	30	1.75 (0.30)	1.14–2.28	30	1.84 (0.38)	1.10–2.57	30	1.75 (0.44)	0.97–2.77
Response time/correct [sec]	30	1.78 (0.38)	1.20–2.79	30	1.70 (0.30)	1.14–2.23	30	1.81 (0.37)	1.09–2.47	30	1.72 (0.43)	0.98–2.74

*NSSI* non-suicidal self-injury, *n* count, *sec* seconds, *SD* standard deviation, *No-stress* task performance before stress induction, *stress* task performance following stress induction

**Fig. 6:**
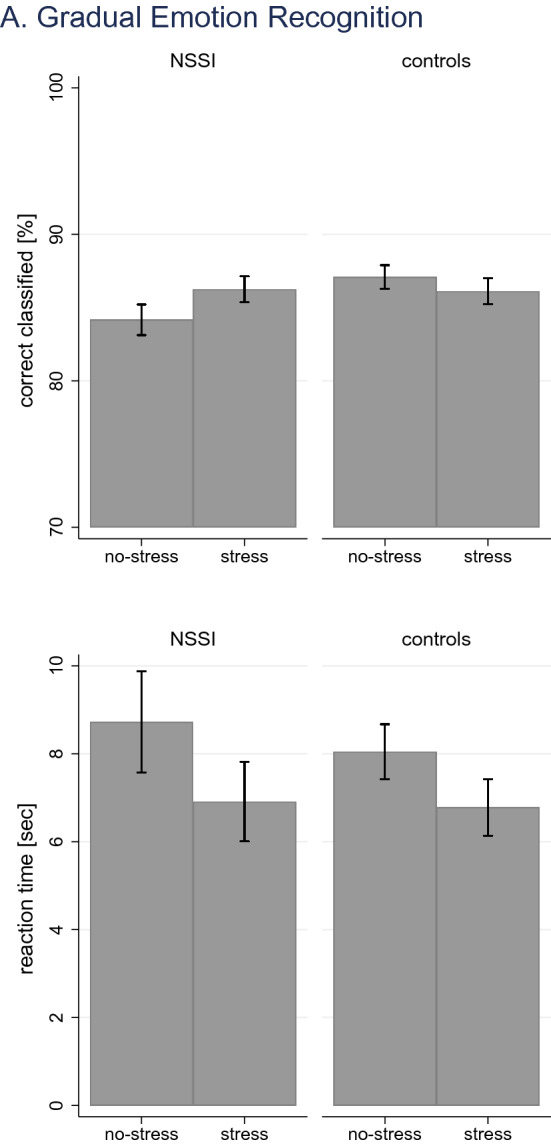
Effects of stress induction on gradual emotion recognition; illustrated are findings on correct responses and reaction time

Regarding the recognition of gradually expressed emotions, there were no effects on correct response ([*n*]: *χ*^2^_(3)_ = 2.05, *p* = *0.562;* [%]:*χ*^2^_(3)_ = 2.15, *p* = *0.541*). Morph time ([all]: *χ*^2^_(3)_ = 65.82, *p* < *0.0001*) and morph time when correctly classifying emotion ([correct]: *χ*^2^_(3)_ = 77.81, *p* < *0.0001*) showed significant effects. There were no significant effects of GROUP ([all]: (*χ*^2^_(1)_ = 0.72, *p* = *0.398*); [correct]: (*χ*^2^_(1)_ = 0.68, *p* = *0.410*), but STRESS ([all]: (*χ*^2^_(1)_ = 63.11, *p* < *0.0001*); [correct]: (*χ*^2^_(1)_ = 74.75, *p* < *0.0001*). Contrasts showed that following stress induction, morph time decreased on average by 1.54 s [all] (95% CI: – 1.92; – 1.16), and [correct] 1.52 s (95% CI: – 1.87; – 1.18), respectively, independent of group. Morph intensity ([all]: *χ*^2^_(3)_ = 66.10, *p* < *0.0001*) and morph intensity when correctly classifying emotion ([correct]: *χ*^2^_(3)_ = 78.42, *p* < *0.0001*) showed significant effects. There were no significant effects of GROUP ([all]: (*χ*^2^_(1)_ = 0.71, *p* = *0.398*); [correct]: (*χ*^2^_(1)_ = 0.68, *p* = *0.411*), but STRESS ([all]: (*χ*^2^_(1)_ = 63.40, *p* < *0.0001*); [correct]: (*χ*^2^_(1)_ = 75.36, *p* < *0.0001*). Contrasts showed that following stress induction, the required morph intensity decreased on average by 9.58% [all] (95% CI: – 11.94; – 7.23), and [correct] 9.49% sec (95% CI: – 11.63; – 7.35), respectively, independent of group. Response time ([all]: *χ*^2^_(3)_ = 141.11, *p* < *0.0001*) and response time when correctly classifying emotion ([correct]: *χ*^2^_(3)_ = 120.87, *p* < *0.0001*) showed significant effects. There were no significant effects of GROUP ([all]: (*χ*^2^_(1)_ = 1.18, *p* = *0.278*); [correct]: (*χ*^2^_(1)_ = 1.85, *p* = *0.173*), but STRESS ([all]: (*χ*^2^_(1)_ = 139.82, *p* < *0.0001*); [correct]: (*χ*^2^_(1)_ = 118.61, *p* < *0.0001*). Contrasts showed that following stress induction, the response time decreased on average by 0.77 s [all] (95% CI: – 0.90; – 0.64), and [correct] 0.62 s (95% CI: – 0.73; – 0.51), respectively, independent of group. Findings are illustrated in Fig. 6.

**Fig. 7:**
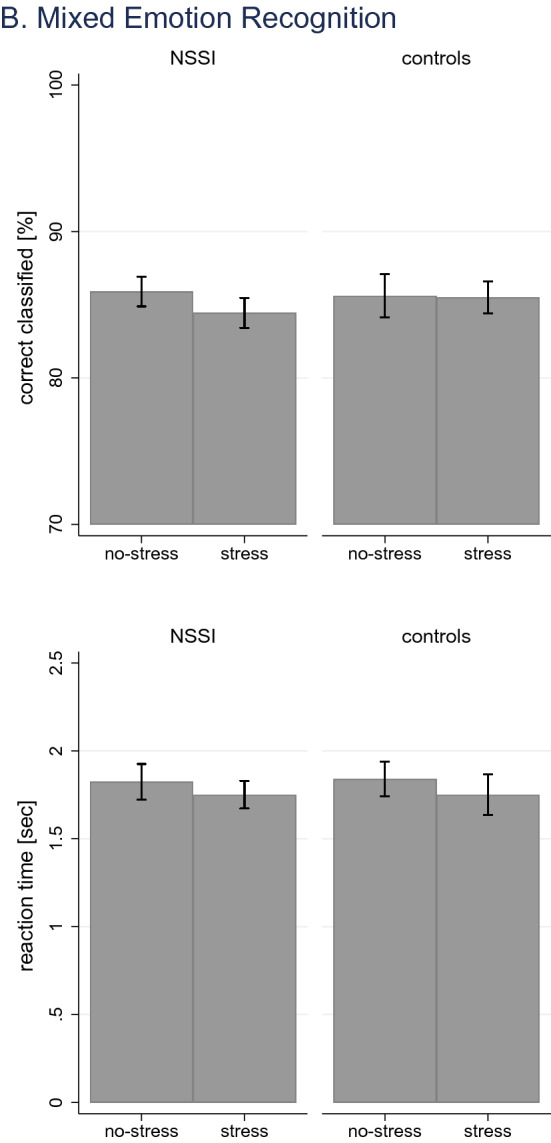
Effects of stress induction on mixed emotion recognition; illustrated are findings on correct responses and reaction time

Analyses of data on the recognition of mixed expressed emotions showed similar findings. There were no effects on correct response ([*n*]: *χ*^2^_(3)_ = 1.67, *p* = *0.644;* [%]: *χ*^2^_(3)_ = 1.18, *p* = *0.759*). Models on reaction time ([all]: *χ*^2^_(3)_ = 4.44, *p* = *0.218*) and reaction time when correctly classifying emotion ([correct]: *χ*^2^_(3)_ = 4.98, *p* = *. 0.173*) showed no significant model fit, despite significant main effects of STRESS ([all]: (*χ*^2^_(1)_ = 4.39, *p* = *0.036*); [correct]: (*χ*^2^_(1)_ = 4.87, *p* = *0.027*). Effects were of small magnitude illustrating a decrease in reaction time following stress induction of [all] – 0.08 s (95% CI: – 0.16; – 0.01), and [correct] – 0.09 s (95% CI: – 0.16; – 0.01), respectively. Findings are illustrated in Fig. 7. Analyses by emotion revealed no specific effects of emotion (Table 3).

**Table 3:** Descriptive statistics by task outcome for mixed emotions, by emotion, group and time

Mixed Expressed Emotions	Adolescents with NSSI						Adolescents without NSSI					
	No-stress			Stress			No-stress			Stress		
	*n*	Mean (SD)	Range	*n*	Mean (SD)	Range	*n*	Mean (SD)	Range	*n*	Mean (SD)	Range
Anger/fear												
Correctly classified [*n*]	30	16.30 (2.25)	11–20	30	16.07 (2.30)	11–20	30	16.00 (2.39)	10–19	30	15.93 (2.12)	11–20
Correctly classified [%]	30	81.5 (11.23)	55–100	30	80.33 (11.52)	55–100	30	80.00 (11.96)	50–95	30	79.67 (10.98)	55–100
Response time [sec]	30	1.82 (0.43)	1.28–2.69	30	1.76 (0.33)	1.01–2.34	30	1.83 (0.41)	1.12–2.51	30	1.78 (0.51)	0.87–3.10
Response time/correct [sec]	30	1.78 (0.42)	1.20–2.69	30	1.68 (0.33)	0.98–2.26	30	1.78 (0.38)	1.10–2.48	30	1.77 (0.50)	0.81–3.04
Happiness/anger												
Correctly classified [*n*]	30	17.53 (1.74)	13–20	30	17.33 (1.71)	13–20	30	17.73 (2.16)	10–20	30	17.87 (1.76)	12–20
Correctly classified [%]	30	87.67 (8.68)	65–100	30	86.67 (8.54)	65–100	30	88.67 (10.82)	50–100	30	89.33 (8.78)	60–100
Response time [sec]	30	1.82 (0.37)	1.19–2.74	30	1.79 (0.38)	1.04–2.49	30	1.85 (0.42)	1.05–2.65	30	1.78 (0.45)	0.94–2.69
Response time/correct [sec]	30	1.78 (0.37)	1.16–2.68	30	1.75 (0.39)	1.06–2.62	30	1.83 (0.40)	1.07–2.63	30	1.75 (0.44)	0.93–2.73
Happiness/Fear												
Correctly classified [*n*]	30	17.70 (1.60)	15–20	30	17.27 (1.66)	13–20	30	17.63 (2.13)	11–20	30	17.50 (2.00)	12–20
Correctly classified [%]	30	88.50 (8.00)	75–100	30	86.33 (8.30)	65–100	30	88.17 (10.63)	55–100	30	87.50 (9.98)	60–100
Response time [sec]	30	1.83 (0.44)	1.17–3.05	30	1.70 (0.31)	1.15–2.30	30	1.84 (0.40)	1.03–2.72	30	1.70 (0.46)	0.89–2.71
Response time/correct [sec]	30	1.78 (0.42)	1.17–3.09	30	1.67 (0.31)	1.08–2.33	30	1.81 (0.42)	0.97–2.72	30	1.64 (0.45)	0.87–2.75

*NSSI* non-suicidal self-injury, *n* count, *sec* seconds, *SD* standard deviation, *No-stress* task performance before stress induction, *stress* task performance following stress induction

Further, in secondary analyses, addressing the association between relative change in emotion recognition following stress induction and BPD symptom severity (indexed by the number of fulfilled BPD criteria and the BSL-23), we found no significant correlations between symptom severity and changes in gradual or mixed emotion recognition following stress induction (all *p* > *0.05*).

## Discussion

This is the first study investigating the effects of psychosocial stress (and the respective psychobiological reactivity) on facial emotion recognition in adolescents across the BPD spectrum. Following contemporary theories on stress-induced impairments in emotion recognition [11], adolescents with NSSI were expected to show subtle impairments in emotion recognition under psychosocial stress. However, these expectations were not confirmed. Adolescents with NSSI were as accurate in emotion recognition as adolescents without NSSI, regardless whether emotion recognition was assessed before or after stress induction.

Adolescents with and without NSSI showed stress-induced disturbances that are commonly observed in psychosocial stress studies [61]. Most of these disturbances were comparable between adolescents with and without NSSI (e.g., changes in stress and cortisol levels). There were, however, some notable differences. Adolescents with NSSI showed more psychological disturbances (e.g., lower positive and higher negative affect), more endocrinological disturbances (e.g., lower α-amylase levels) and greater physiological disturbances (e.g., lower HR and higher HRV responses) than adolescents without NSSI, indicating profound alterations in multiple stress systems.

Although the hypothalamus–pituitary–adrenal stress system appeared to be unaltered, there were marked alterations in the sympathetic stress system. Adolescents with NSSI showed similar cortisol levels but lower α-amylase levels than adolescents without NSSI. Adolescents with NSSI also showed lower HR and HRV in response to stress than adolescents without NSSI, indicating stress-induced alterations in the sympathetic and parasympathetic branch of the autonomous nervous system. The stress-induced alterations in the parasympathetic nervous system may have contributed to the affective disturbances of adolescents with NSSI because alterations in HR and HRV are often associated with affective changes in stressful contexts [62–64]. Importantly, we were able to demonstrate that stress-responsiveness differed as a continuous function of BPD severity. These secondary analyses illustrated that as the subjective stress response (dissociation and negative affect) increased, the biological stress response decreased (cortisol and HR) as a function of BPD severity. These findings provide some novel and important insights into BPD pathology and help to elucidate some prior conflicting findings in the literature.

Stress-induced disturbances in adolescents with NSSI have already been reported in a previous study [65], albeit in different stress systems. Adolescents with NSSI differed in cortisol levels but not in HR or affect levels from adolescents without NSSI in that study, whereas adolescents with NSSI differed in α-amylase, heart rate or affect levels but not in cortisol levels form adolescents without NSSI in the present study. Stress-induced disturbances, thus, encompass more than a limited set of confined stress systems in NSSI. Our findings of a continuous moderation of the stress response by BPD severity enable the integration of these, previously conflicting findings. Similar conclusions can be drawn from studies in BPD where stress-induced differences in cortisol, α-amylase, HR and affect have been observed between adults with and without BPD [29, 30, 66–69]. These studies suggest that psychosocial stress alters multiple stress systems in adolescents and adults on the BPD spectrum, thereby leading to a multitude of psychological, endocrinological and physiological disturbances. Here we, to our knowledge, provided first empirical support for a continuous influence of BPD pathology on the spectrum of severity.

Although adolescents with NSSI showed altered stress responses compare to adolescents without NSSI, there were no differences in emotion recognition following stress induction. Adolescents with NSSI recognized all emotions with similar accuracy and similar speed as adolescents without NSSI, regardless whether task performance was assessed before or after the stress induction. There was a general increase in task performance following stress induction. Gradual emotions were recognized at lower intensity levels and with greater speed following stress induction, indicating a stress-induced facilitation of recognition sensitivity and recognition speed. Mixed emotions were also recognized with greater speed following stress induction, indicating once more a stress-induced facilitation of recognition speed. Stress increased recognition sensitivity and recognition speed to a similar extent in adolescents with and without NSSI. Adolescents with NSSI, thus, showed stress-induced improvements rather than stress-induced impairments in emotion recognition. These improvements were at odds with the impairments that had been expected on basis of contemporary theories on emotion recognition under psychosocial stress [11]. However, it might well be plausible that the observed effects are explained by design aspects, resulting from the repeated task performance. Although we implemented two different versions of each task using different stimuli to account for this, we cannot rule out such general practice effect in task performance.

Emotion recognition under psychosocial stress has rarely been investigated in adolescents with NSSI. A previous study investigated emotion recognition in adolescents with NSSI throughout a mood-induction procedure with disturbing movies [70], which may also elicit some sort of psychosocial stress [71]. The mood-induction procedure had, however, no effects on recognition sensitivity and recognition accuracy during the processing of gradual emotions. Adolescents with NSSI recognized all emotions at similar intensity levels and with similar speed as adolescents without NSSI, regardless whether task performance was assessed before or after the stress-provoking mood manipulation. The present study revealed similar recognition sensitivity and recognition accuracy in adolescents with and without NSSI following a more stress-provoking manipulation, indicating that it is quite unlikely that stress impairs emotion recognition in NSSI. Studies in BPD support this conclusion. Adults with BPD were as accurate in the recognition of gradual expressions of emotions as adults without BPD, regardless of whether the emotion recognition task was performed before or after the stress-provoking manipulation [29, 30]. The stress provocation led to an improvement rather than impairment of task performance because stress increased recognition accuracy to a similar extent in adults with and without BPD [29]. These studies clearly show that psychosocial stress has, if at all, no deleterious effects on emotion recognition in adolescents and adults on the BPD spectrum.

The findings of the present and previous studies challenge the contemporary view that the BPD spectrum is characterized by stress-induced impairments in emotion recognition [11]. Stress is thought to alter arousal levels in adolescents and adults on the BPD spectrum in way that differentially affects the processing of emotions at low and high intensity levels. Low intensity emotions are believed to be recognized with higher than normal accuracy and high intensity emotions are believed to be recognized with lower than normal accuracy, indicating subtle rather than frank impairments in emotion recognition across the BPD spectrum. However, the findings of the present and previous studies clearly refute these propositions [29, 30, 70]. Adolescents and adults on the BPD spectrum show neither stress-induced impairments during the processing of low intensity emotions nor stress-induced impairments during the processing of high intensity emotions. Stress-induced impairments in emotion recognition are, thus, less prevalent across the BPD spectrum than commonly assumed.

Although the findings of the present and previous studies need to be replicated and extended in future studies, the findings may already be of great interest for researchers and clinicians working with adolescents and adults on the BPD spectrum. Researchers may be interested to know that psychosocial stress—although eliciting a clearly altered psychobiological response—is probably less relevant for emotion recognition impairments across the BPD spectrum than hitherto assumed [11], implying that research should focus on other factors than psychosocial stress to explain emotion recognition deficits in adolescents and adults on the BPD spectrum. Clinicians may be interested to note that stress-induced emotion recognition deficits are less common across the BPD than previously thought [5, 13], implying that treatment should focus on other factors than psychosocial stress to improve emotion recognition deficits in adolescents and adults on the BPD spectrum.

### Electronic supplementary material

Below is the link to the electronic supplementary material.

Supplementary file1 (DOCX 21 kb)

Supplementary file2 (PDF 98 kb)

Supplementary file3 (PDF 97 kb)

Supplementary file4 (PDF 102 kb)

Supplementary file5 (PDF 96 kb)

## Appendix

### Diagnostic criteria for NSSI according to DSM-5 [3]

Criterion A: engagement in NSSI on 5 or more days in the past year.

Criterion B: the expectation that NSSI will (1) provide relief form a negative feeling or cognitive state; and/or (2), resolve an interpersonal difficulty; and/or (3) induce a positive feeling state.

Criterion C: NSSI is associated with either (a) interpersonal problems or negative thoughts or emotions immediately prior to NSSI, and/or (b) preoccupation with NSSI that is difficult to control, and/or (c) frequent thoughts about NSSI, even when it is not acted upon.

Criterion D: NSSI is not socially sanctioned or restricted to picking a scab or nail biting.

Criterion E: NSSI causes clinically significant distress or interference in interpersonal, academic, or other important areas of functioning.

Criterion F: NSSI does not occur only in the context of psychosis, delirium, or substance use/withdrawal and is not better accounted for by another psychiatric disorder or medical condition.

## Footnotes

1 Oxygenation of the prefrontal cortex was continuously recorded using an 8-channel functional fNIRS system (OctaMon, Artinis Medical Technologies, BV, The Netherlands). fNIRS data are not reported in the present manuscript.
