# Supplementary material for: Altered psychobiological reactivity but no impairment of emotion recognition following stress in adolescents with non-suicidal self-injury
Source: Eur Arch Psychiatry Clin Neurosci. 2022 Oct 6;273(2):379–95. doi: 10.1007/s00406-022-01496-4 (PMC10070238; doi:10.1007/s00406-022-01496-4)
Supplement: Supplementary file 1 — Supplementary file1 (DOCX 21 kb) [file 406_2022_1496_MOESM1_ESM.docx]

**Supplementary Material**

**Reasons for exclusion and dropout**

A total of n = 180 consecutive patients were screened. Of these n = 143 (79.4%) were excluded for: being male (n = 31); no up-to date clinical diagnostics were available (n = 8); not meeting criteria for NSSI (n = 28); not interested in study participation (n = 10); acute suicidality, substance abuse or psychotic symptoms (n = 20); BMI below or above the defined threshold (n = 9); did not respond after initial contact (n = 8); taking glucocorticoid medication (n = 8); were pregnant (n = 1); reporting a neurological or endocrinological disorder (n = 4); did not provide written informed consent (n = 16). N = 37 patients were invited for participation in the study, of which n = 7 dropped-out. N = 4 did not show up to their study appointment; n = 3 discontinued participation during testing for acute headache (n = 1) or severe dissociative symptoms (n = 2). A total of n = 63 adolescents without NSSI were initially contacted for participation in the study, of which n = 32 were excluded after initial screening for not being interested in further participation (n = 10); intake of glucocorticoid medication (n = 3); reporting of a neurological or endocrinological disorder (n = 2); showing psychiatric symptoms (n = 16); or not responding any further (n = 1). N = 31 adolescents without NSSI were included in the study, of which n = 1 dropped out during the experiment. The final sample comprised n = 30 adolescents with NSSI and n = 30 adolescents without NSSI.

**Supplementary Table 1: Complete ECG (HR and HRV) Data by GROUP and Segment of TIME**

| **Segment** | **Group** | **N** | **Duration** | **% Artefact free** | **N IBI** |
| --- | --- | --- | --- | --- | --- |
| Baseline 1 | Patients | 29 | 299.20 (0.44) | 99.48 (0.88) | 388.97 (49.83) |
|  | Controls | 30 | 297.76 (8.32) | 98.57 (3.42) | 388.03 (68.76) |
| Baseline 2 | Patients | 29 | 307.73 (2.18) | 99.57 (0.54) | 407.21 (53.25) |
|  | Controls | 30 | 305.31 (6.53) | 99.15 (1.82) | 407.50 (53.31) |
| GradEmo A | Patients | 30 | 321.17 (71.69) | 99.37 (0.82) | 406.90 (104.43) |
|  | Controls | 29 | 311.39 (40.07) | 99.22 (1.57) | 401.76 (58.03) |
| MixEmo A | Patients | 30 | 229.16 (23.95) | 99.39 (0.41) | 296.43 (50.39) |
|  | Controls | 28 | 229.66 (24.05) | 99.17 (1.12) | 298.75 (54.91) |
| Postline 1 | Patients | 30 | 305.76 (1.81) | 99.65 (0.18) | 392.93 (49.75) |
|  | Controls | 28 | 305.32 (1.22) | 99.12 (1.13) | 391.36 (53.01) |
| TSST Preparation | Patients | 30 | 312.37 (11.98) | 98.02 (7.24) | 425.37 (78.69) |
|  | Controls | 28 | 323.93 (33.79) | 99.16 (1.77) | 486.32 (89.33) |
| TSST Free-Speech | Patients | 29 | 299.70 (1.46) | 97.86 (9.42) | 455.00 (88.61) |
|  | Controls | 28 | 298.18 (4.09) | 98.66 (3.65) | 516.11 (101.72) |
| TSST Mental Arithmetic | Patients | 29 | 299.32 (0.66) | 97.88 (8.64) | 445.00 (84.43) |
|  | Controls | 28 | 298.29 (4.31) | 98.33 (4.72) | 489.50 (107.32) |
| GradEmo B | Patients | 29 | 263.70 (53.40) | 98.03 (5.57) | 311.72 (78.05) |
|  | Controls | 28 | 260.70 (35.75) | 99.09 (1.02) | 322.21 (55.57) |
| MixEmo B | Patients | 28 | 222.84 (19.08) | 97.29 (9.19) | 263.11 (49.96) |
|  | Controls | 28 | 223.77 (28.23) | 98.72 (1.53) | 274.93 (59.64) |
| Postline 2 | Patients | 28 | 305.07 (1.87) | 97.24 (10.91) | 366.00 (70.09) |
|  | Controls | 28 | 303.69 (3.88) | 98.95 (1.79) | 377.79 (54.30) |

N: data used for analyses; duration: mean duration of the respective recording segment and standard deviation in seconds; % artefact free: percentage of artefact free inter-beat intervals; IBI: mean number and standard deviation od inter-beat intervals used for analyses
