# Supplementary figures and images for: Altered psychobiological reactivity but no impairment of emotion recognition following stress in adolescents with non-suicidal self-injury
Source: Eur Arch Psychiatry Clin Neurosci. 2022 Oct 6;273(2):379–95. doi: 10.1007/s00406-022-01496-4 (PMC10070238; doi:10.1007/s00406-022-01496-4)

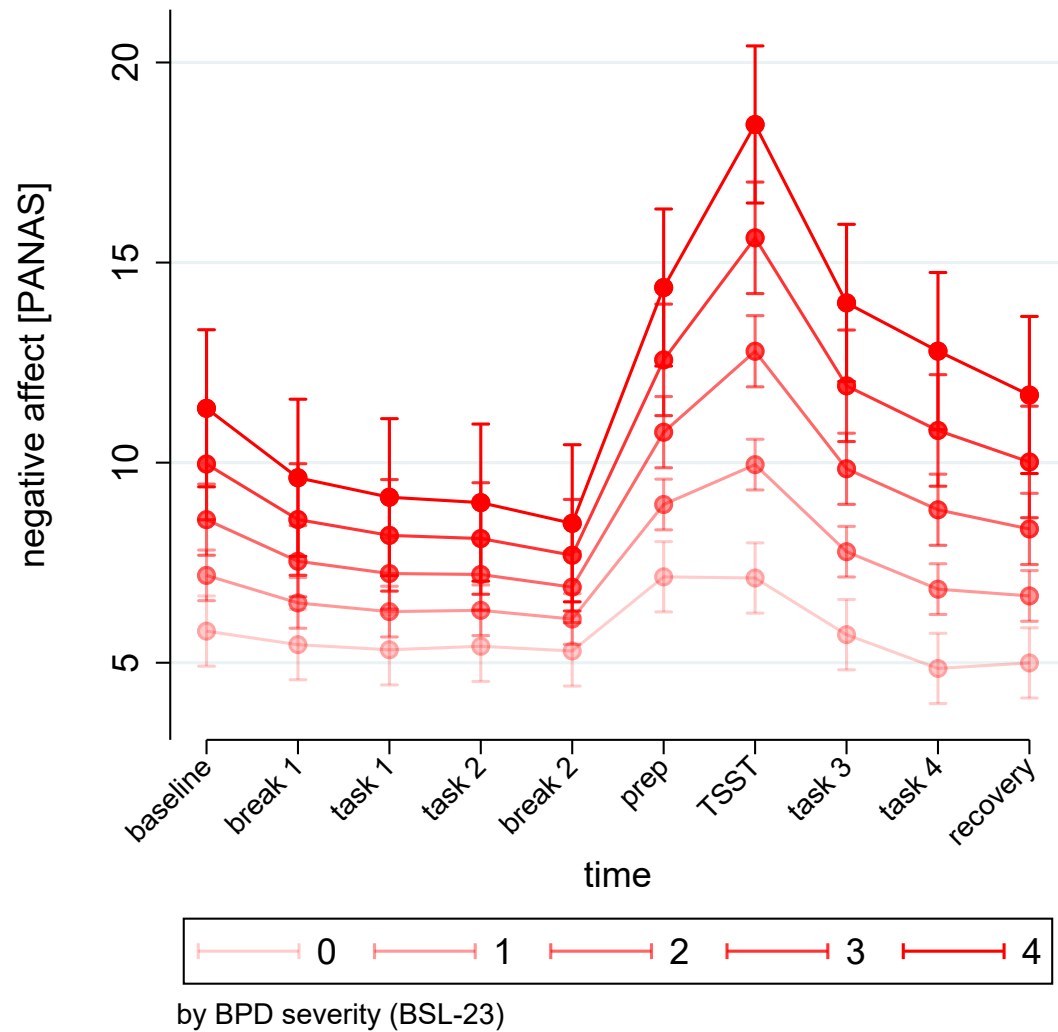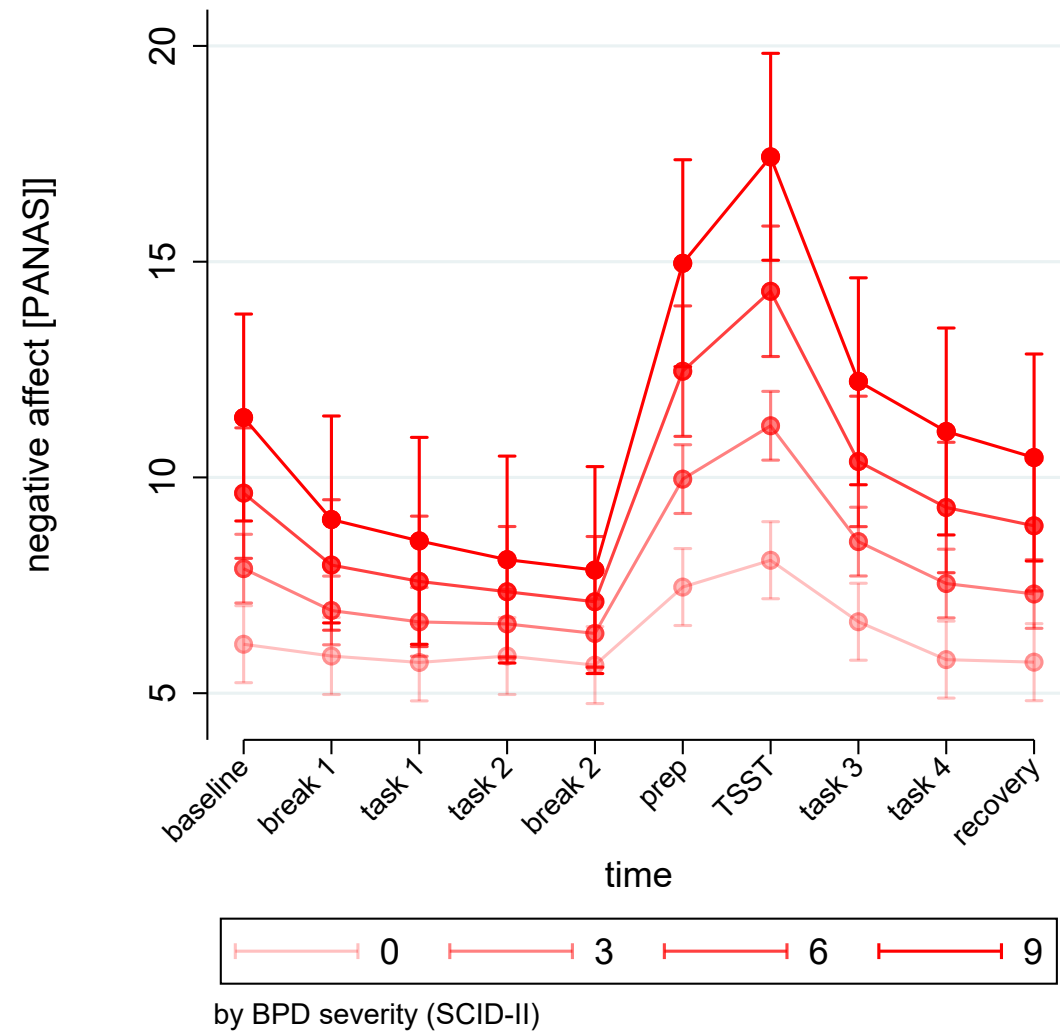

Supplement: Supplementary file 2 — Supplementary file2 (PDF 98 kb) [file 406_2022_1496_MOESM2_ESM.pdf]

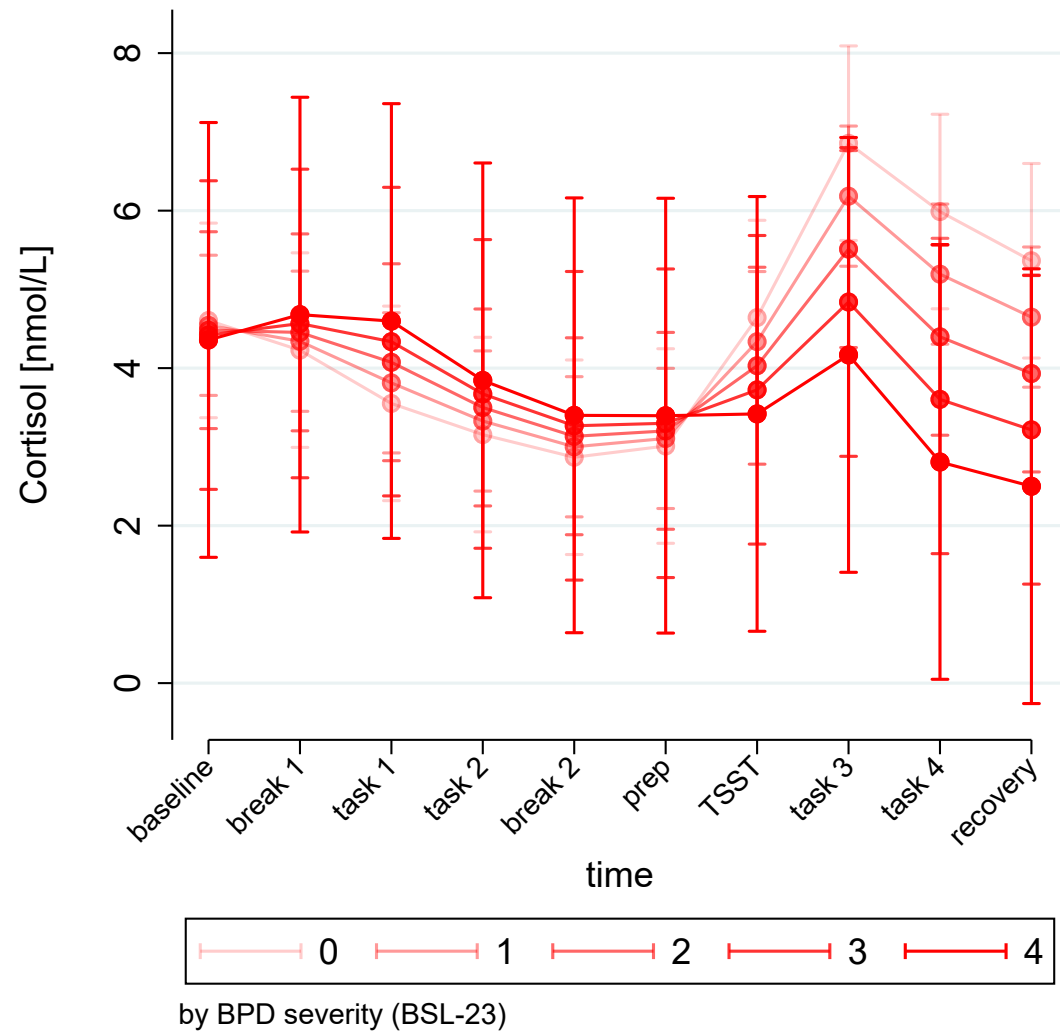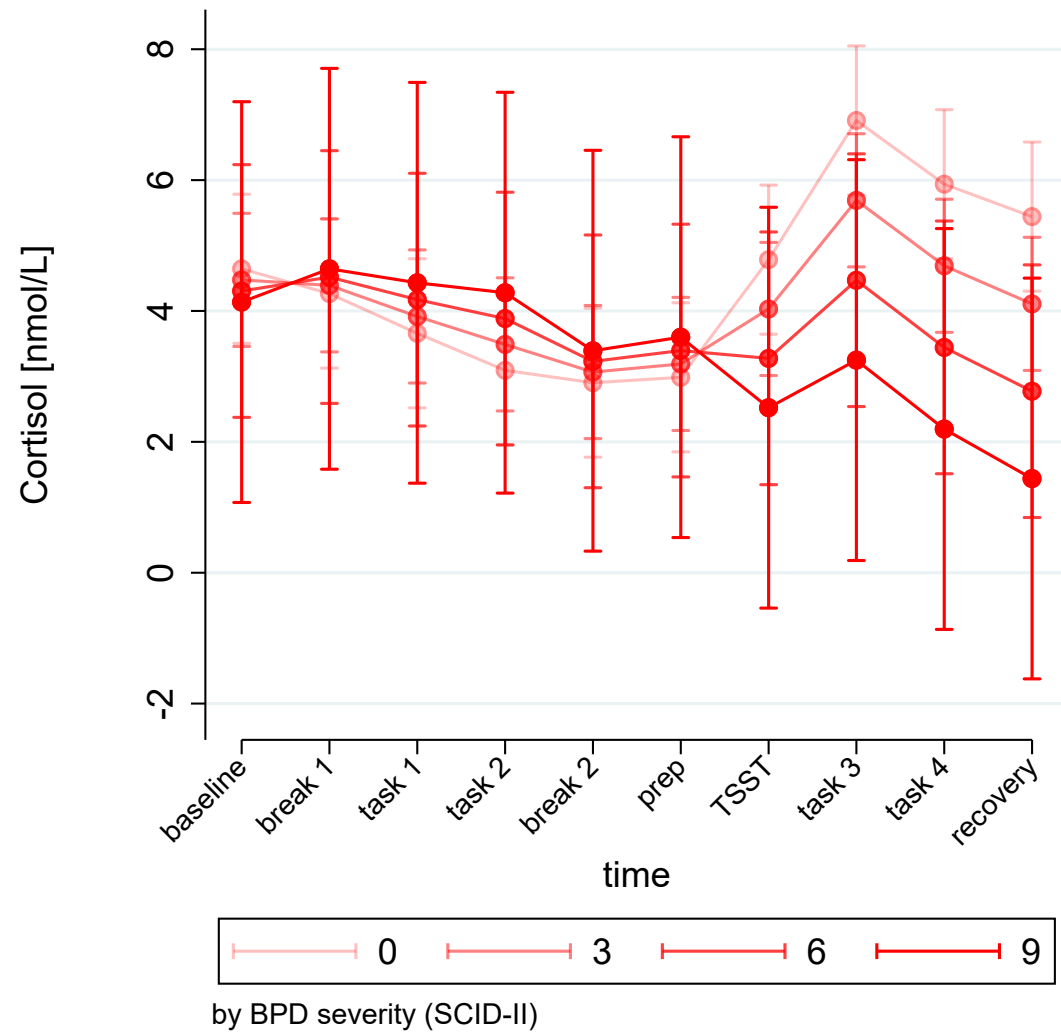

Supplement: Supplementary file 3 — Supplementary file3 (PDF 97 kb) [file 406_2022_1496_MOESM3_ESM.pdf]

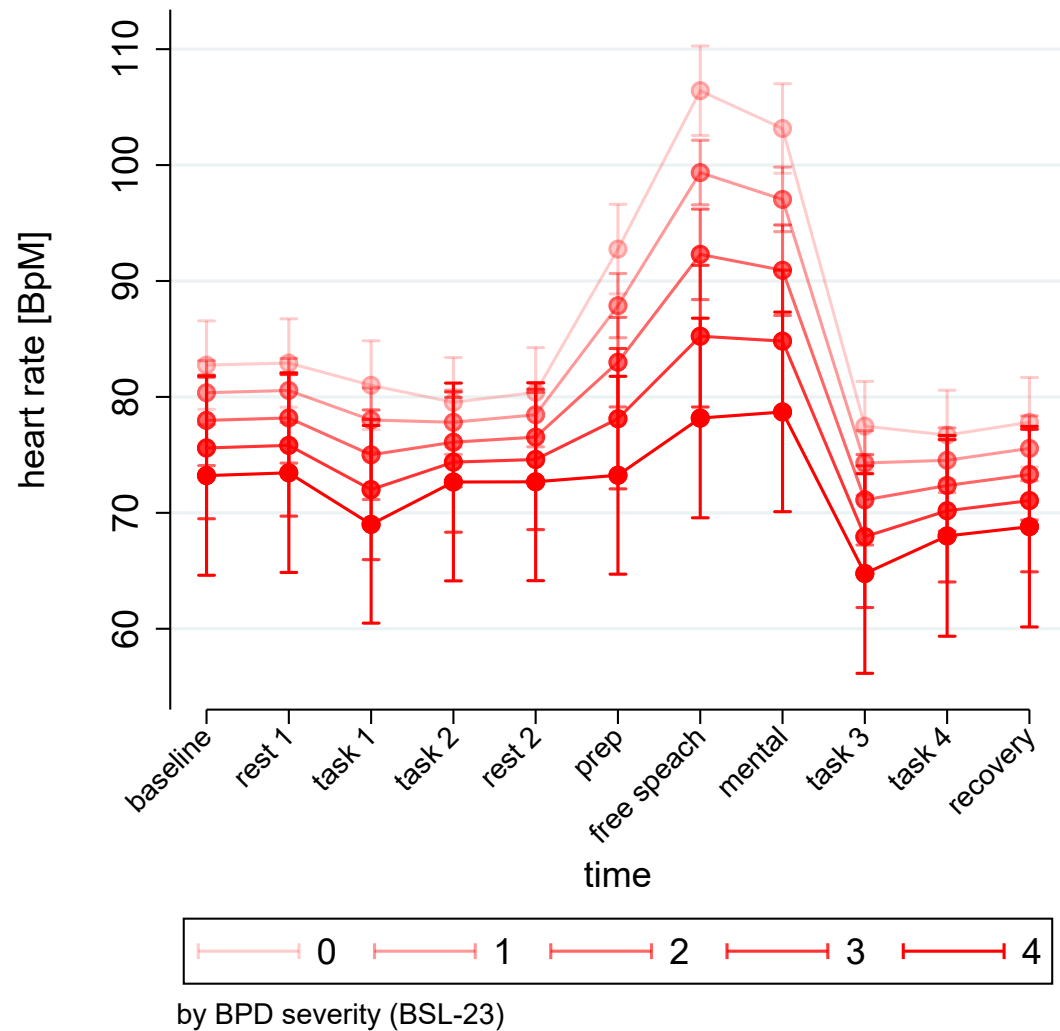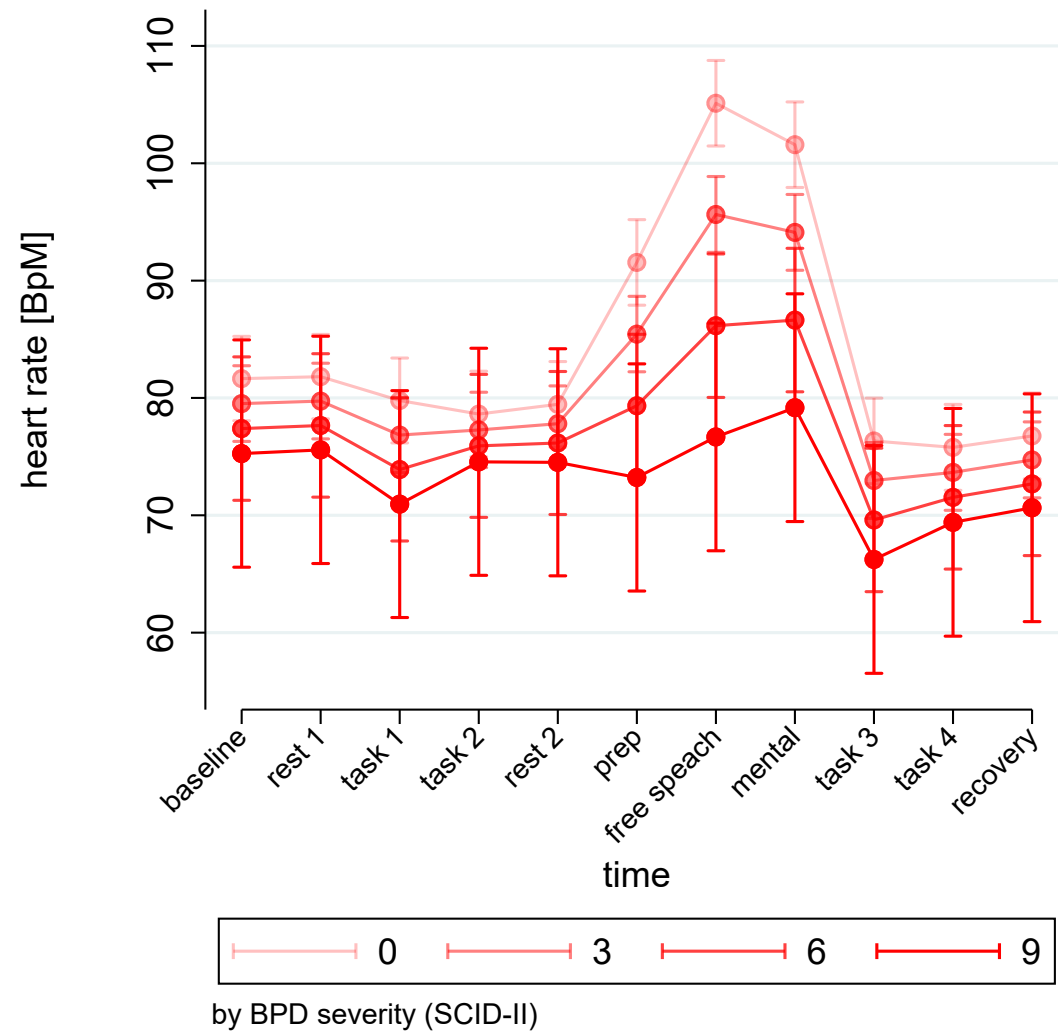

Supplement: Supplementary file 4 — Supplementary file4 (PDF 102 kb) [file 406_2022_1496_MOESM4_ESM.pdf]

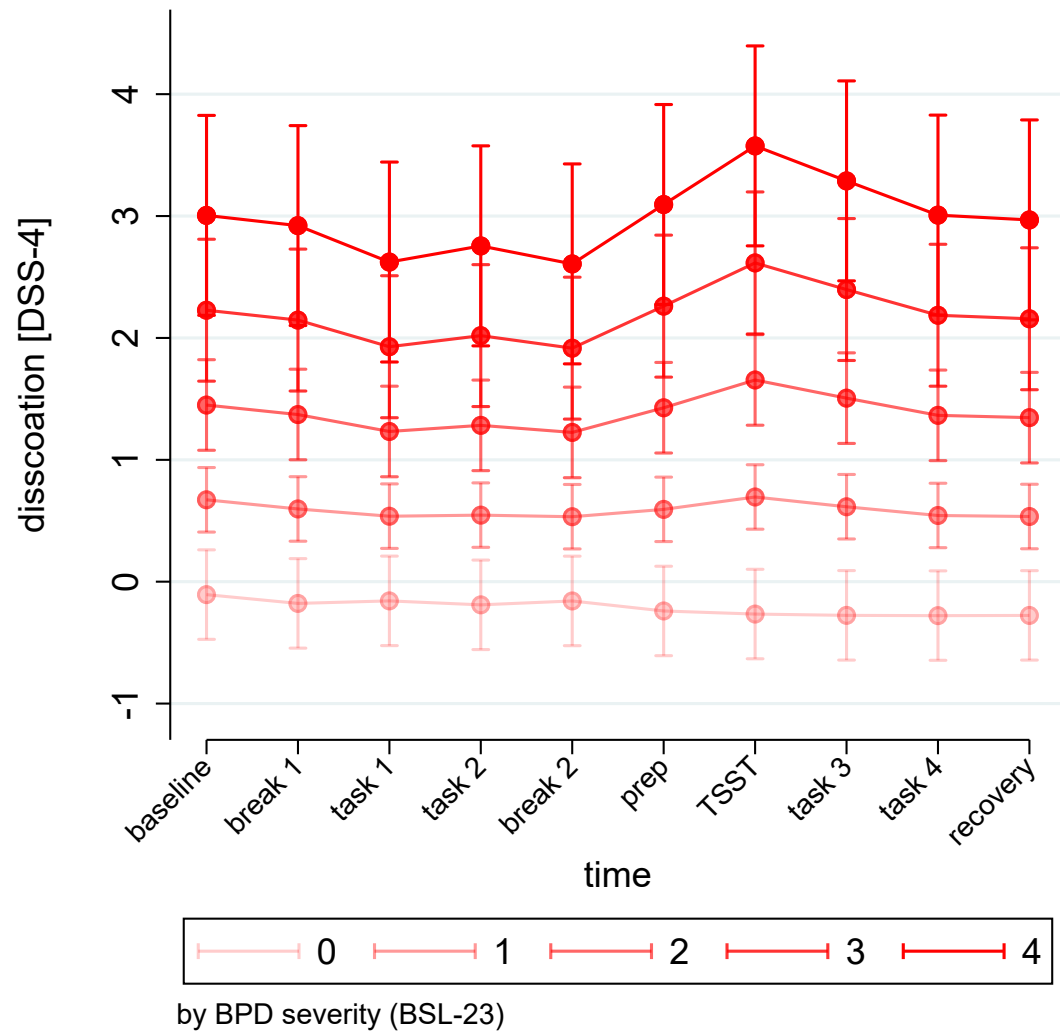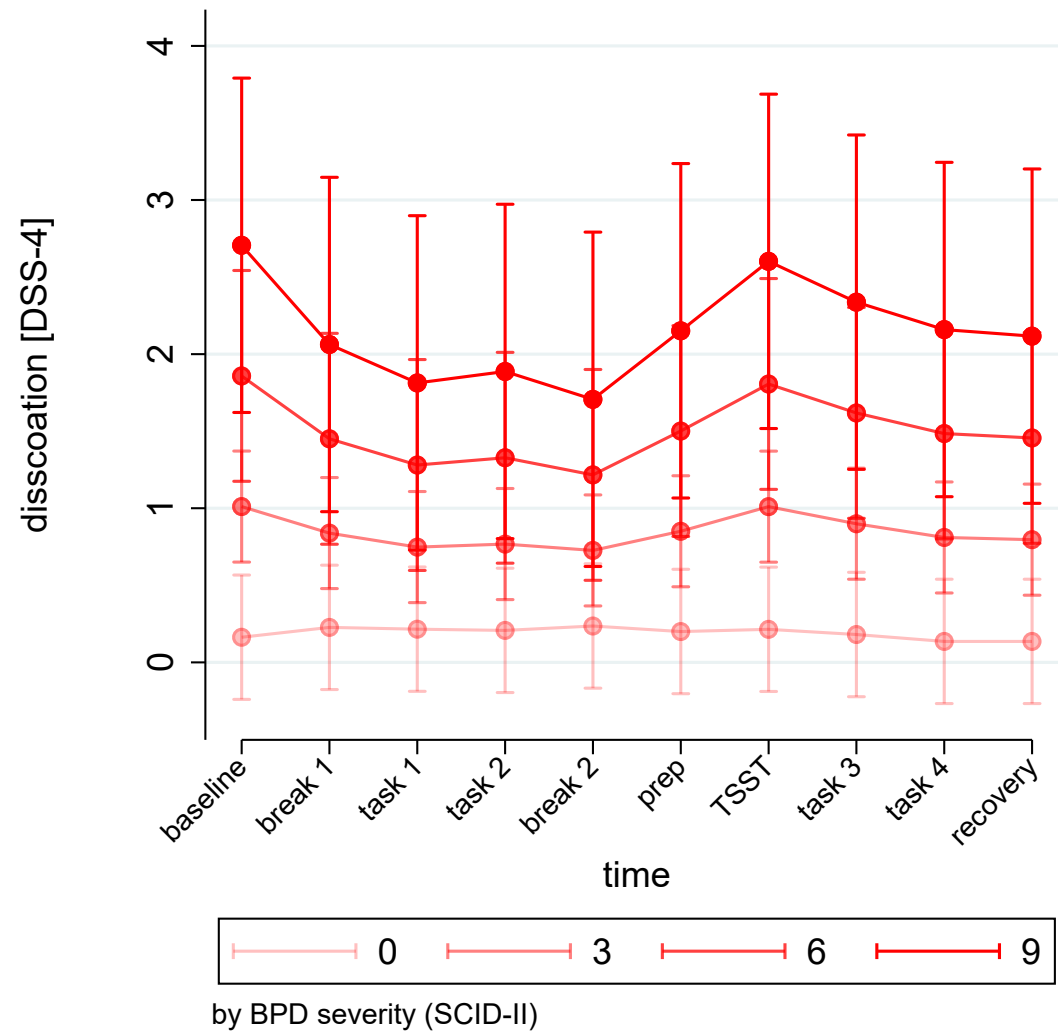

Supplement: Supplementary file 5 — Supplementary file5 (PDF 96 kb) [file 406_2022_1496_MOESM5_ESM.pdf]
